# Mental health as a prerequisite for functioning as optimally as possible in old age: A phenomenological approach

**DOI:** 10.1002/nop2.698

**Published:** 2021-01-10

**Authors:** Samal Al Gilani, Lina Tingö, Annica Kihlgren, Agneta Schröder

**Affiliations:** ^1^ Nutrition and Physical Activity Research Centre Faculty of Medicine and Health Örebro University Örebro Sweden; ^2^ School of Health and Sciences Faculty of Medicine and Health University Health Care Research Centre Örebro University Örebro Sweden; ^3^ Nutrition Gut Brain Interactions Research Centre Faculty of Medicine and Health Örebro University Örebro Sweden; ^4^ Faculty of Medicine and Health Örebro University Örebro Sweden; ^5^ Department of Health Sciences Faculty of Medicine and Health Science NTNU – Norwegian University of Science and Technology Gjøvik Norway

**Keywords:** mental health, older adults, optimal functionality, phenomenological analysis, qualitative

## Abstract

**Aim:**

To describe the impact of mental health on the ability to function optimally among older adults experiencing mental health issues.

**Design:**

This study had a descriptive qualitative design.

**Methods:**

Six older females with a Hospital Anxiety and Depression Scale (HADS) score of ≥8 on either of the subscales (depression or anxiety) participated in individual interviews. All data were analysed using a phenomenological approach influenced by Giorgi.

**Results:**

The phenomenological analysis led to a structured synthesis comprising the following three themes: (a) life situations affecting mental health, (b) consequences of mental health in everyday life and (c) strategies for maintaining mental health.

## 1 INTRODUCTION

Population ageing is increasing at a high rate, and it is estimated that between 2015–2050 the percentage of older adults in the world will almost double from 12%–22% (World Health Organization, 2017). In total numbers, that is an increase from 900 million to two billion older adults over the age of 60 (World Health Organization, 2017). According to Statistics Sweden (2018), it is estimated that by the year 2028, older adults over the age of 80 will have increased by 50% (Statistics Sweden, 2018). For the first time in history, most individuals will reach old age and live beyond their sixties. Older adults over the age of 60 are important to society; they are important to their families, are active in volunteering and are still members of the workforce (World Health Organization, 2017). However, with increased age comes the risk of health issues. According to the World Health Organization, health issues among older adults can be neurological disorders, diabetes, hearing loss and mental disorders, among others (World Health Organization, 2017). It has further been confirmed that the increase in individuals reaching high age will inevitably be accompanied by increased rates of physical and mental ailments (Oliver et al., 2014; Rechel et al., 2013).

Maintained mental health is an important aspect of growing older and both older adults themselves and healthcare professionals often under‐identify mental health issues. Furthermore, the issue of mental health and its stigma tends to lead to animosity towards seeking the professional help that is needed (World Health Organization, 2017). Previous research suggests that both of these factors are important for the older adult's ability to experience optimal functionality (Algilani et al., 2014); however, there is a need to further explore the aspect of mental health in relation to the older adult's ability to function as optimally as possible (Algilani et al., 2016a).h

The prevalence of mental illness among older adults in Sweden is increasing. Today approximately 20% of the older population over the age of 65 suffer from mental health issues and it is estimated that the percentage will increase to 25% within a near future, which will make it one of the largest endemic diseases in Sweden (National Board of Health & Welfare, 2018). According to the National Board of Health and Welfare, cross‐sectional studies indicate a prevalence of 5%–15% for depression and 6%–12% for anxiety (National Board of Health & Welfare, 2018). Both depression and anxiety are known to affect an individual's ability to function (Westerhof & Keyes, 2010) and can also negatively affect an older adult's quality of life, daily living and everyday activities (Locke et al., 2015). Among older adults, depression alone is an important public health issue and a major cause of disability (Ferrari et al., 2013).

Mental illnesses are seldom revealed in primary care (Bland, 2012) as older adults deny having feelings of anxiety or depressive symptoms and prefer to discuss insomnia, irritability and agitation in addition to other somatic complaints (Bland, 2012). Furthermore, older adults with mental health issues generally avoid seeking psychiatric help because of the costs, geographical distance to the care facility, feelings of shame, mistrust of mental health providers and not knowing where to turn (Brenes et al., 2015). In addition, previous research has pointed out deficiencies in the collaboration between the different organizations, the older adults and their next of kin (Swedish Association and of Local Authorities and Regions, 2012). This indicates that older adults living with mental health issues possibly are at great risk of remaining an invisible group as far as health care and society in general are concerned (Andersson and Josephson, 2014). A person‐centred approach, which also can serve as a tool to promote self‐care, is needed for healthcare professionals to better meet and care for older adults adequately (Astin & Closs, 2007; Dale et al., 2012) so as to allow for the older adult to function as optimally as possible in everyday life.

The concept of optimal functionality has previously been explored as a way of capturing subjectively experienced factors that are of importance for the individual to function as optimally as possible in their current season of life. Optimal functionality has been described as a concept weighing together body‐related factors (e.g. physical well‐being), self‐related factors (e.g. mental well‐being) and external factors (e.g. environmental conditions) (Algilani et al., 2014). We further performed a focus group‐based study with older adults to extend the qualitative understanding of optimal functionality (Algilani et al., 2016a). Interestingly, there was a lack of discussion about the influence of mental aspects on optimal functionality. Concerning the barriers that previously have been described as hindering older adults from reaching out with their mental health concerns, the current study was initiated to learn more about the impact of mental health on older adults’ optimal functionality. Hence, the aim of this study was to describe the experience of mental health and its impact on the ability to function as optimally as possible among older adults with mental health issues.

## 2 METHOD

This study used a descriptive qualitative design to capture the experience of mental health and its impact on the ability to function as optimally as possible among older adults with mental health issues.

### 2.1 Selection of study participants

A total of one hundred older adults aged ≥65 from an existing study cohort (Östlund‐LagerstrÖm et al., 2016b) took part in a follow‐up study that included several self‐completion questionnaires. Among these instruments, data from the Hospital Anxiety and Depression Scale (HADS) were collected.

Hospital Anxiety and Depression Scale is a well‐used instrument for evaluating mental anguish and distress in hospital and primary care settings and in the general population (Snaith, 2003; Zigmond & Snaith, 1983). The instrument has been tested with good validity and reliability (Bjelland et al., 2002; Hermann, 1997) and is constructed as a self‐completion questionnaire consisting of two subscales measuring mental distress (Snaith, 2003). The complete scale contains 14 questions that can either be divided into two independent subscales, that is anxiety and depression, or be used combining all items into a global score of mental distress. The two scales can be interpreted as follows: 0–7 scores (normal), 8–10 (mild distress), 11–14 (moderate distress) and 15–21 (severe distress) (Snaith, 2003).

Out of 100 questionnaires sent out to this cohort of older adults, 70 completed questionnaires were returned. Seven older adults displayed scores that correspond to an elevated level of distress, that is a score of ≥8 (score range: 8–14) on either the depression and/or the anxiety subscale and were hence asked to participate in the study. All gave their consent to participate; however, one of them later decided not to participate. Finally, six participants, all female, were enrolled in the study (Table 1).

**TABLE 1:** Demographic data of study participants

Participant^a^	Age (years)	HADS score^b,c^ (Depression)	HADS score^b,c^ (Anxiety)
Participant 1	73	14	12
Participant 2	71	4	8
Participant 3	70	10	14
Participant 4	70	8	8
Participant 5	69	6	9
Participant 6	71	4	8

^a^ All study participants were female.

^b,c^ Scores ≥8 indication for depression^b^/anxiety^c^.

### 2.2 Data collection

The first author in this study contacted the eligible study participants by telephone, gave them information about the study and also set up a date and place for the interview. Prior to every interview, the study participants were given oral and written information about the aim of the study, the voluntary nature of their participation, their right to withdraw, the safekeeping of the collected data and confidentiality. The open interview method described by Dahlberg et al. (2008) was used in order to capture the participants’ experience of the studied phenomenon. One main question was posed to initiate the interviews:

Describe a situation where your mental health has an impact on your experience of functioning as optimally as possible. Follow‐up questions were asked if needed, for example: Can you elaborate this, please? or What did you mean by that?

All interviews lasted 45–90 min each and were conducted by the first author, who is a psychiatric nurse. The interviews were audio‐recorded and all were held at the university, except one that was held in the person's home. An authorized secretary transcribed all six interviews verbatim.

### 2.3 Analysis

The transcribed data were analysed using a phenomenological approach influenced by Amadeo Giorgi (Giorgi, 1997). Phenomenology aims to capture and describe the “life‐world” of the study participants. In phenomenology, it is important for the researcher to avoid interpretations of the study participants’ narrated experiences and to present the life‐world as it actually appears to the respondent. The researcher therefore needs to leave pre‐understandings behind and find an attitude that is more objective to let the world show itself as it is (Nyström & Dahlberg, 2001).

Pre‐understanding should be put in “brackets” meaning that the participant's experience of a phenomenon should not be affected by the researcher (Nyström & Dahlberg, 2001). To facilitate the bracketing in this study, the pre‐understanding of the first author (who performed the interviews) was reflected upon, taken into consideration and discussed with the research group (Hamill, 2010). The analysis was initiated after the last interview had been transcribed.

The process of analysis followed the principles outlined by Giorgi (1997) and was carried out in collaboration between the authors of this study. In the first step, all transcribed data were read through several times to obtain an overall understanding of the data as a whole. In the second step, the data were broken down into “meaning units” to reveal and identify information about the phenomenon. In step three, the meaning units were organized, reformulated and rewritten into transformed meaning units with the disciplinary language of choice. By doing so, the content of the meaning units could be expressed from a scientific perspective. The transformed meaning units were then organized in order to reveal patterns and variations in the data. Finally, in step four, the meaning units were condensed to obtain a structured synthesis.

Throughout the analysis, the transformed and organized meaning units were compared to the original meaning units to ensure that the descriptions were consistent and exhaustive. Discussions about this process were ongoing within the research group throughout the entire analysis process.

### 2.4 Ethics

This study was approved by the Uppsala Regional Ethics Review Board (dnr. 2012/309). The study participants received information about the aim of the study, and written consent was obtained prior to the interviews. The study participants were also informed that the collected data would be kept in a safe place and that confidentiality was guaranteed.

## 3 RESULTS

The older adults included in this study describe several factors related to mental health as affecting their ability to function as optimally as possible. A structured synthesis revealed a tripartite result where the following themes came forward: (a) life situations related to mental health, (b) consequences of mental health issues in everyday life and (c) strategies for maintaining mental health as affecting their ability to function as optimally as possible. Henceforth, these three themes are referred to as (a) life situations (related to mental health), (b) consequences (of mental health issues) and (c) strategies (for maintaining mental health).

The synthesis further revealed that these three themes are intricately linked and all appear to have an effect on one another: for example, the older adults in this study repeatedly described life situations that had consequences in everyday life, which in turn led to the development of strategies for maintaining good mental health. Importantly, however, the three themes were not always sequential; for example, the absence of preventive strategies could give rise to negative situations leading to negative consequences, but they did not necessarily follow the same order for all individuals at all occasions. This indicates that the three themes are closely linked but do not follow any specific sequential order.

**FIGURE 1:**
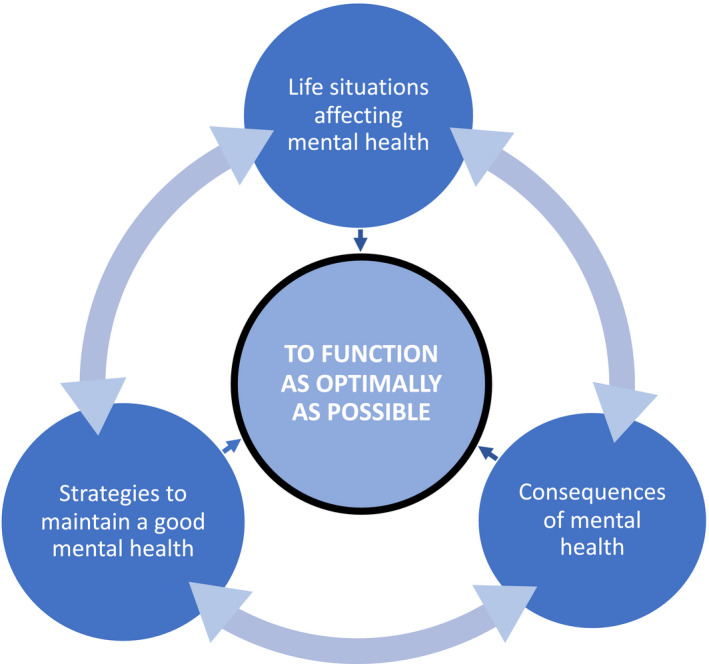
The synthesis of the structured experience comprising three components

Furthermore, the notion of functioning as optimally as possible was found to be affected by all three themes (i.e. life situations, consequences and strategies) together or separately. Negative situations in life may for example affect the older adult's ability to function as optimally as possible, just as suffering from the consequences of one's mental health issues and strategies (or lack thereof) can impact on optimal functionality. See Figure 1 for a synthesized structure of the three themes. The results will be further presented below, followed by quotations.

### 3.1 Life situations affecting mental health

A majority of the study participants discussed physical concerns as a “life situation” that could adversely impact their mental well‐being and ability to function optimally. The ageing body itself poses a challenge:

…well, physical things as well. You notice that your body changes, which affects you… I think this thing with the body [changing] you could say is tough, that you feel that your body… It's not like when you were younger, but the [poor physical] health affects you.” (P3).

The older adults described that changes in bodily functions, including sleep deprivation, gastrointestinal issues, impaired vision, balance problems, headaches, migraines and anosmia all had a negative impact on their mental health and hence their ability to function optimally:

“…I have a problem with my stomach and… I worry, it affects everything. That I have to run to the toilet often and I’m a bit worried… (P1)

Furthermore, the health of family members, that is having concerns about their poor health and one's own health and fear of becoming ill, played a significant role as life situations. Deaths in the family and being concerned about family members after one's own death were also further related to poor mental health.

The older adults also spoke of the importance of the relationships among family members’ relations, including poor relationships with and between family members. Living far away from family and friends was described as an obstacle to experiencing good mental health:

And I think that this also affects you, since the world will be a bit smaller. … I have my best friends, they don't live in Örebro either, they live elsewhere and it's like… You can't just go over there and say hi and sit and chat for a while but… And that means that my husband and I are very much more dependent upon each other than we were before. (P3).

Having a poor relationship with your spouse, going through a divorce and being reminded of painful events or unpleasant incidents between family members affected one's mental health, which in turn had an impact on one's ability to function optimally:

Yes, it makes me sad that … I don't ask for much, just a little kindness from him towards her. For him to feel she is his sister. Although we, you could say, do have some sibling love, they keep on quarrelling… That's something I’ve done with my brother, but we do get together, there's no real problem. But we say whatever … that was then. He was a bit eager and rowdy as a child. Many children are quite calm … we were lively. We had a lot space… so that was good, but… No, it's our children. So we sometimes say, well…what a pity that we got such children, no peace with them. (P5).

Shortcomings in the upbringing of one's children and one's own upbringing were dwelled upon and described as situations that could affect one all the way into old age. Remembering negative experiences from one's upbringing affected everyday life situations. Difficult childhoods and negative life experiences caused long‐term negative effects on one's mental health and were described as leading to introversion. Moreover, encouragement was experienced as having a positive effect on mental health, while the lack of encouragement led to anger later in life. Feeling supported and spending time with family and friends were highly appreciated and had positive impacts on mental health.

In addition, life situations about social aspects were described as important. For example, living in a small town and being close to activities and events was experienced as positive, whereas the lack of social interaction was not:

But when I still lived where I lived before, in a smaller town, [with] friends nearby, we lived a fun life and were out and danced and so like… When you were out doing such things you forgot about it and had a good time (P1).

Not getting anything in return when being emotionally generous and feeling that one's benevolence was taken advantage of resulted in poor mental health, as did feelings of inadequacy, such as not being good enough, feeling inferior and being under pressure due to external demands. To be needed was however a great contributing factor to the experience of good mental health, as was having the company of a pet.

Some external factors were described as producing stress. These included traffic situations, not knowing how to use computers and the rapid development of modern technology. All of these had a negative impact on mental health and the ability to function as optimally as possible. Modern technology in general was described as a life situation that affected mental health, as it could give rise to feelings of powerlessness. Concerns about family members and who would take over the family business, or just worrying about the future in general, were experienced as having an impact. In addition, worries about one's finances and financial independence had an impact on mental health:

But finances also have an impact. If you are retired like I am, with a very small pension, it has an impact… Mentally, in the long run, it does. (P2)

The fear of becoming dependent on others had an impact on mental health affecting the ability to function as optimally as possible. This included being afraid of ending up in a nursing home, becoming physically dependent on others and becoming bedridden. Healthcare experiences were described as having an impact on mental health and one's ability to function as optimally as possible. The older adults’ mental health and mood were also influenced by the seasons and the weather, which in turn impacted on their ability to function as optimally as possible:

Yes, I get low. I have trouble getting going with things sometimes. … Going to the movies or something like that… Then again, we do manage to find something I guess… I am not as happy… I think that I’m extremely influenced by the weather. (P4)

Mental health was also described as being dependent upon the time of day, as evening darkness gave rise to feelings of insecurity that had an impact on mental health. The older adults were very concerned about the politics of the world and the functioning of society. Worrying about the future and the world around them was described as having an impact on their life situation.

### 3.2 Consequences of mental health issues

The consequences of mental health issues were described as affecting the opportunity to function as optimally as possible in several ways. Psychotropic pharmaceuticals were considered helpful and were expected to improve one's mental health:

I mean when I felt so bad then… I took what was called Celexa (Citalopram) at the time, it like takes away… It helped me then of course, when I felt… It evens things out, there isn't any of this… And sometimes I can long for it…so that you don't have this anxiety all the time, but…”. (P3)

Sleep deprivation was considered to be closely related to mental health. The fatigue that it results in has an impact on everyday life, as it makes it difficult to go outdoors. Poor mental health in general led to a loss of initiative to do things.

Worrying was described as an aspect of poor mental health that presents an obstacle to living life as you wish, for example worrying about things and people, and worrying about one's own health. Worrying about the health of family members, such as children and grandchildren, gave rise to feelings of powerlessness. Moreover, brooding and having existential thoughts gave rise to anxiety and sadness and became a consequence of poor mental health that affected the older adults’ opportunity to function as optimally as possible. Brooding was described as leading to a downward spiral of worry that hindered the older adults from functioning optimally and had an impact on their mental health:

…that it's kind of running through my mind, you sit there … When I get worried I get really worried. Then I’m at a loss…” (P3)

Likewise, stress was described as a spiral that produced more stress. Just growing old per se was described as stressful and impacted one's ability to function optimally. In addition, experiencing stress gave rise to other issues such as annoyance, distress, worsened joint problems and deteriorated physical condition, which impacted life and could be described like this:

Yes, I feel a little stressed. I am perhaps a little irritable in the morning. That is, I’m not… I don't want to talk to anyone then, like…no. (P4)

…easy to be stressed and… When you should be on your way and such… Yes, it is sort of an extra dose of stress, heart palpitations…” (P6)

Furthermore, feelings of anger were a consequence that could affect the older adults’ life situations. Having an unsatisfactory relationship with a spouse, not being able to take the initiative for a divorce and staying in a bad marriage because of fear of loneliness were described as leading to poor mental health. Loneliness could also have an impact on the mental health of the older adults. They described that loneliness was accompanied by negative thoughts and brooding, which were experienced as obstacles to their ability to function as optimally as possible.

### 3.3 Strategies for maintaining good mental health

The older adults also talked about strategies to feel better, that is things to alter their mental health in a positive manner, which in turn had an impact on their ability to function as optimally as possible.

Diversion was described as a strategy for maintaining mental health. To divert attention from difficult things, strategies such as travelling, being present (in the moment), engaging in mindfulness and concentrating on other things were mentioned. Such strategies could prevent negative thoughts from arising at difficult times. One participant said their strategy for diversion in this way:

…it's always in the back of your mind so when you are sitting alone the thoughts come back. And mindfulness is so good, then I can use it, put on the tape and listen to it. Then the thoughts go away… I get going.” (P5).

Another strategy was to ask for support, as it contributed to good mental health. Talking to a family member and having someone who was going through the same thing were described as comforting. Having the ability to take the initiative and doing things were also strategies used to attain better mental health. By taking the initiative and doing things, the older adults did not brood and thus diverted their anxiety to something else. Having pets to keep one company was another strategy for improving one's mental health, as was engaging in physical activity:

But then I myself have a cat. It is of course also important. Yes…[to] cuddle with. It is kind of like…you feel a bit upset when you wake up in the night…you pet the cat and become calm. That's the way…animals are… Earlier when I was growing up I had a dog, and that was of course good. Yes…mmm. Having a pet I think plays a big role in people being able to feel good.” (P4)

Having time for oneself was described as a strategy that gave a sense of freedom and increased the experience of mental wellness. Earning your own money was also associated with feelings of being free and thus increased the chances of maintaining good mental health, affecting the ability to function as optimally as possible. Taking an interest in something, such as genealogy, gardening or being active in an organization, was another strategy mentioned for maintaining mental health.

Determination was another strategy mentioned, as it helped one to get through the day: not giving up, being more optimistic and not hiding one's head under the blanket despite emotional darkness. On the other hand, loss of determination made one “surrender,” which led to a decreased zest for life:

You have to make the best of what is available. It is not possible to bury yourself completely … even though there are dark days. (P5)

Having the strategy of feeling gratitude, joy and contentment for what you have and the strategy of making the most of your situation both improved mental health, as did accepting one's life as it is. Finally, experiencing adversity was described ambiguously as both something that could make one stronger and wiser but also something that could lead to mental illness.

## 4 DISCUSSION

### 4.1 Findings

The phenomenological approach (Giorgi, 1997) used in this study generated a tripartite structured synthesis describing how mental health affects older adults’ ability to function as optimally as possible. Among older adults with mental health issues, the experience of mental health and its impact on the ability to function as optimally as possible were characterized by the three themes of life situations (related to mental health), consequences (of mental health in everyday life) and strategies (for maintaining good mental health).

Life situations may be related to physical concerns, family, social aspects or external factors. As a physical concern, the ageing body itself created anxiety that had an adverse impact on mental health and the ability to function optimally. This is consistent with a study that showed a direct association between functional limitations and anxiety (Goncalves et al., 2011). Mobility is among the most important functions to maintain independence and to participate in various forms of activities with others (Avlund et al., 2004). Along with ageing, older adults might experience multiple losses that may threaten their sense of significance (Nolan et al., 2011).

The life situations that were described as affecting mental health in this study are varied and individually experienced. This reaffirms the subjectivity of the experience of mental health and its influence on the ability to function optimally and is also in line with Barker's Tidal model for recovery (Barker & Buchanan‐Barker, 2011). This model states that people's experiences are unique and that they are alone in them. In this context, it is clearly important to learn about a person's life situation and what it actually means for the individual (Barker & Buchanan‐Barker, 2011).

Furthermore, all the older adults spoke about the importance of family, including life situations that involved their family and different concerns about their family members. This indicates that the family has a major impact on the older adult's mental health status. With a lack of close relationships, a sense of alienation can develop where loneliness can lead to negative memories and thoughts (From, 2007). The sense of belonging may have a protective role against loneliness in older adults living at home (Prieto‐Flores et al., 2011). Engagement in the family, mainly children and grandchildren, can structure and control everyday life for the older person, instead of the older person focusing on illness and disability (Vik et al., 2008).

External factors such as stress, finances and the risk of becoming dependent had an impact on their mental health, since increased dependency affects one's self‐image (Hammarström & Torres, 2010). The findings in this study also show that sleep deprivation, worrying about things, existential thoughts, brooding and loneliness were all consequences that had considerable impact on the older adults’ mental health, which in turn affected their ability to function as optimally as possible.

The older adults in this study considered pharmaceuticals helpful, which can be an indication of a previous or present impaired health status. A study from Canada states that older adults with a poor health status use prescribed drugs more intensively (Carrie et al., 2006). It is however important to note that the over‐prescription of medication in older adults can increase the chances of negative health issues and increase healthcare costs (Onder et al., 2014).

Developing strategies to maintain good mental health was something that all the older adults described. Two effective strategies mentioned by most of them were sleeping and engaging in physical activity. Getting enough sleep led to improved mental health according to the older adults in this study. This indicates that if they did not get enough sleep, increased brooding could develop, which would affect mental health adversely. This is in line with a study showing that a depressive mood was associated with decreased deep sleep time (Smagula et al., 2015). One strategy to improve sleep is to listen to music, which is perceived in research studies as an effective, non‐invasive and easy method for older adults to improve their sleeping habits (Chan et al., 2009; Jespersen et al., 2015). That physical activity is a strategy that can be used to maintain good mental health has also been confirmed by research which indicates that dance (Hwang & Braun, 2015), tailored exercise programmes (Hoffmann et al., 2015) and plain regular physical activity (Zhang & Yen, 2015) can be beneficial for mental health and can reduce depressive symptoms (Hwang & Braun, 2015; Hoffman et al., 2015; Zhang & Yen, 2015).

The older adults in the current study helped to fill the gap that rose in a previous study by the Algilani et al., (2016a) where the concept of optimal functionality, that is the notion of functioning as optimally as possible, was extended and deepened (Algilani et al., 2016a). In the previous study of optimal functionality, all aspects in the structure of the concept were discussed by the older adults except for the one about mental aspects (Algilani et al., 2016a). This is interesting as mental aspects have previously been described as a predictor of optimal functionality (Algilani et al., 2014). With the findings of the current study in mind, it may be suggested that the mental aspect is intricately linked to all the major themes of the structure of optimal functionality. This might be an explanation for mental health not appearing clearly in the previous studies of optimal functionality (Algilani et al., 2014; Algilani et al., 2016a). A factor that to such an extent is intertwined with all other aspects of optimal functionality is easily missed, as it is not clear where one ends and the other begins.

When conducting research influenced by phenomenology, the objective is to take on an attitude of phenomenological reduction. For the researcher, this primarily means that one must bracket prior knowledge about the phenomenon being researched. This means holding back one's existential index and only taking in what is given as it is given (Giorgi, 1997), or, as Dahlberg et al. (2008) describe, use the newer and more positive idea of bridling. As the first author of this study, who conducted all interviews, is a nurse specializing in psychiatric care and the phenomenon in question pertained to mental health, this was a delicate matter. In order to obtain phenomenological reduction, her pre‐understanding was reflected upon and discussed in the research group for the purpose of bridling (Dahlberg et al., 2008) and bracketing (Giorgi, 1997). Reaching phenomenological reduction is a matter of how well it has been reflected upon throughout the research process (Burns & Grove, 2003; Graneheim & Lundman, 2004). Yet, whether or not a sufficient level has been reached can always be questioned.

It is of importance to discuss the trustworthiness of the findings of the study. The authors conclude that the findings can be extended to other Western contexts involving older adults over the age of 65. To further ensure trustworthiness, the selection of study participants, the data collection process and the process of analysing data have been thoroughly described in the methods section (Graneheim & Lundman, 2004). Reliability is another important factor to discuss and was aimed for by meticulously describing all processes in the study; however, it can be very difficult to recreate all the steps in every process in order to get the same results. Perhaps if other researchers attempt to recreate the study, it will be difficult to retain the same results due to different pre‐understandings. Nevertheless, all processes have been described in detail so as to make it possible for other researchers to redo and repeat the study process (Polit & Hungler, 1999).

### 4.2 Limitations

There are a few methodological aspects that should be taken into consideration when interpreting this study's results. For example, the fact that all of the older adults participating in this study were women may have had an impact on the findings. The one male that was contacted and initially planned to participate in the study withdrew. It is possible that there are even greater barriers for men than for women to discuss mental health issues. This has been suggested in previous studies where the male respondents associate mental health problems with shame, engage less in mental health services and seek out mental health professionals less than females do (Gouwy et al., 2008; Pattyn et al., 2015). In addition, that the study participants were in a narrow age range (69–73 years) can be seen as a factor limiting credibility. This was a result of applying our inclusion criteria (i.e. receiving a score of ≥8 on the questionnaire of HADS). In addition, a larger initial population (i.e. *N* > 100) may have resulted in a more heterogeneous study sample. However, there is also an opportunity that mental health issues actually occur less frequently among the oldest individuals of our initial population. Westerhof and Keyes (2010), for example, have shown in a previous study that mental illness was indeed frequent among older adults, but not among the oldest old.

### 4.3 Implications for nursing practice

This findings of this study have several implications for clinical recommendations and contribute important knowledge to nursing practice.

The problems with stigma surrounding mental illness can make older adults hesitant or unwilling to seek help. When older adults such as the ones who participated in this study seek contact with the healthcare system, one of the challenges for the healthcare professionals will be how to best address the multifaceted experience of mental health as described in this study. Healthcare professionals in general and nurses in particular are bound by law to offer care that is personalized and based on the preferences of the individual they care for.

One of the main findings of the study concerns the importance of family. The nurse in clinical practice hence needs to be aware of this and, if needed, involve the family to actively take part in order to help the patient on the way towards improved mental health and thus optimal functionality. However, personalized care and person‐centred care are key points in helping patients towards improved mental health, so the nurse also needs to be aware that not all patients may want or need the support of family. Financial issues surfaced as another important factor leading to impaired mental health, which in turn affected optimal functionality. This is thus an important factor for the nurse in clinical practice to be aware of in order to personalize the patient's care. Sleep and physical activity were described as important factors and also as strategies for maintaining mental health and, in turn, optimal functionality. When nurses encounter older adults with mental health issues in clinical practice, it is crucial to raise awareness that strategies such as changing sleep patterns and engaging in physical activity are important for achieving mental health and optimal functionality.

## 5 CONCLUSION

This study adopted a phenomenological approach to contribute to a deeper understanding of the phenomenon of mental health issues and their impact on older adults’ ability to function as optimally as possible. The findings show that mental health affects older adults’ ability to function optimally by impacting their life situations, consequences thereof and the development of strategies to maintain good mental health in everyday life. These three themes, together or separately, affected the older adults’ ability to function as optimally as possible. When older adults with mental health issues seek contact with health care, this information will be essential for meeting the multifaceted needs of this patient group.

## 6 ETHICS APPROVAL AND CONSENT TO PARTICIPATE

This study was approved by the Uppsala Regional Ethics Review Board (dnr. 2012/309). Oral and written information were given to the participants who agreed to participate signed the written consent for participation.

## CONFLICT OF INTERESTS

The authors report no conflict of interest.

## AUTHOR CONTRIBUTIONS

AK designed the study. SA and LOL made the data collection and the initial analysis of the collected data. Each step of the analysis was then scrutinized and discussed by all authors. SA and LOL head the responsibility to write the manuscript. Furthermore, all authors made critical revisions to the manuscript, and read and approved the final manuscript.

## CONSENT TO PUBLISH

The stakeholders have given their consent to publish the data obtained.
